# Phenomenological and existential contributions to the study of erectile dysfunction

**DOI:** 10.1007/s11019-021-10029-0

**Published:** 2021-06-09

**Authors:** Chris A. Suijker, Corijn van Mazijk, Fred A. Keijzer, Boaz Meijer

**Affiliations:** 1 Department of Urology, University of Groningen, University Medical Center Groningen, Groningen, The Netherlands; 2 Faculty of Philosophy, University of Groningen, Groningen, The Netherlands

**Keywords:** Erectile dysfunction, Phenomenology, Merleau-Ponty, Perception, Body schema, Existentialism

## Abstract

The current medical approach to erectile dysfunction (ED) consists of physiological, psychological and social components. This paper proposes an additional framework for thinking about ED based on phenomenology, by focusing on the theory of sexual projection. This framework will be complementary to the current medical approach to ED. Our phenomenological analysis of ED provides philosophical depth and illuminates overlooked aspects in the study of ED. Mainly by appealing to Merleau-Ponty’s *Phenomenology of Perception*, we suggest considering an additional etiology of ED in terms of a weakening of a function of sexual projection. We argue that sexual projection can be problematized through cognitive interferences, changes in the ‘intentional arc’, and modifications in the subject’s ‘body schema’. Our approach further highlights the importance of considering the ‘existential situation’ of patients with ED. We close by reflecting briefly on some of the implications of this phenomenological framework for diagnosis and treatment of ED.

## Introduction

Erectile dysfunction (ED) is a disorder that is characterized by the inadequacy of acquiring or maintaining a penile erection that is sufficient for satisfactory sexual performance (Yafi et al. 2016). ED is a highly common disorder, affecting 19–25% of males younger than 40 years old, according to a study in Israel (Heruti et al. 2004), and 30% of males older than 40 years, according to a study in Europe (Corona et al. 2010). The difficulty with or impossibility of sexual intercourse affects the patient and his partner(s) in negative ways. ED can give rise to a diminished self-esteem, a different sense of masculinity, existential problems and problems within relationships (Tomlinson and Wright 2004; Yafi et al. 2016).

ED has been the focus of several theoretical and empirical approaches. One such approach is the psychoanalytical approach, which focuses on unresolved sexual issues in the unconsciousness of the patient (Freud 1963/1997). More common in contemporary practice is the mixed or biopsychosocial approach (Shamloul and Ghanem 2013; Yafi et al. 2016). The biopsychosocial approach highlights the importance of organic defects, but it also takes psychological and social aspects into account. This is because organic, social and psychological aspects can mutually affect each other in complex (sexual) disorders (Hedon 2003; Fisher et al. 2005; Nelsson et al. 2015). Additionally, one in four patients currently diagnosed with ED is a young man, and in this population, it is likely that most cases of ED result from a complex interaction of several causes (Capogrosso et al. 2013; Yafi et al. 2016).

The biopsychosocial account, like most scientific accounts, has a tendency of objectifying the body and mind, which might lead to a partial neglect of the subjective component of the disorder (Accad 2016). Such neglect may harm successful treatment of the disorder. Research shows that 75% of the males suffering from ED are not being treated (Frederick et al. 2014), and that 75% of this population declares not seeking treatment because they are too embarrassed (Baldwin et al. 2003). Other research shows that 69% of men deny the existence of their ED (De Boer et al. 2005). Objectification of the patient body in current biopsychosocial approaches of ED might contribute to these numbers and the recognized ‘taboo’ that revolves around the discourse of ED (Lyngdorf and Hemmingsen 2004). Additionally, the most available treatment endorsed by the biopsychosocial approach of ED, sildenafil (Viagra©) is not a panacea for the problem: it has a success rate of 63% compared to 33% in the placebo group (Stuckey et al. 2003). Side effects such as headache, flushing, dyspepsia and visual disturbances are very common (Stuckey et al. 2003). Data also indicate that 50–68% of patients stop their (medicative) treatment within a few years (Sundaram et al. 1997; Jiann et al. 2006).

Additionally, the prevalence of ED varies widely from country to country, with a global prevalence of ED estimated at 3–76.5% (Kesler et al. 2019). The biopsychosocial approach, primarily endorsing objective thought, has trouble postulating an explanation for this wide variety in variance, partially because of the fact that there is no ‘objective’ method for diagnosing ED (Yafi 2016). It is very plausible that there are reasons for the global variance in the prevalence of ED that transcend the current biopsychosocial approach and its focus on the objective workings of the body, mind and social environment.

The findings introduced in the previous paragraphs suggest that the current biopsychosocial approach is in need of further development. In this article, we hope to provide a reinforcement of the biopsychosocial approach by developing a phenomenological framework for ED. The goal of this phenomenological framework is to bring approaches that focus on experience closer to the current biopsychosocial account of ED, something that has not been done extensively yet. Generally speaking, phenomenology focuses on the first-person perspective; it is the systematic study of phenomena as consciously experienced (Biemel 2017). As such, it offers a valuable set of conceptual tools for thinking about the subjective component of ED. Phenomenological considerations of ED might break down ‘taboos’ and contribute to stronger doctor–patient relationships, while opening up new and more effective diagnostic and treatment methods.

The framework that we develop in this article is based primarily on Merleau-Ponty’s *Phenomenology of Perception* (1962/2012). This work focuses on subjective perception of the environment, ourselves, our bodies and others. The nature of perception and its relation to ED has not yet been explored in great detail. In the following section (two), we discuss the importance of the field of perception for ED. Section three develops an additional etiology of ED that is able to reinforce the current biopsychosocial etiology. In section four, the implications of the new etiology for the patient will be discussed; section five overviews some of the practical implications for physicians.

## Perception

Phenomenology is commonly considered to be the study of phenomena or appearances. Classic phenomenology in the tradition inaugurated by the German philosopher Edmund Husserl (1859–1938) is strictly limited to the first-person perspective (Smith 2018); it considers how things appear to us or how we experience things. Classic phenomenologists such as Husserl, Martin Heidegger, and Merleau-Ponty maintained that the empirical sciences operate from a distinctively third-person, objectivating perspective called the ‘natural attitude’. They opposed this to the ‘phenomenological attitude’, which does not study any object in particular, but the very objects also studied in science only *qua* subjective appearance as well as the acts of consciousness by which we relate to them.

Today, phenomenology has found fruitful application in nearly all scientific disciplines, including healthcare and psychiatry (Zahavi and Parnas 2002; Ratcliffe 2008; Perier et al. 2013; Fernandez 2020). It can be of use here in particular by exploring how health and illnesses are experienced and perceived by the patient, making it a valuable contribution to any medical doctrine endorsing a patient-central perspective. In this paper, we shall focus almost exclusively on Merleau-Ponty’s work.^1^ Merleau-Ponty’s work has been successfully applied to other fields of the study of sexuality, such as transgender studies (Ahmed 2006; Salamon 2010; Baldino 2015) and feminist studies (Grosz 1993; Heinämaa 2003; Olkowski and Weiss 2010). His works have also been used in studies related to sexual medicine (Pietz 1985; De Preester 2013; Klaeson et al. 2012; Thomas et al. 2015).

In phenomenology, perception is a central focal point in regard of our embodied being and our everyday access to the social world. For instance, perception draws our attention, thereby motivating bodily action (Merleau-Ponty 1962/2012, pp. 216–223 and pp. 315–316). For this reason, some phenomenologists prefer to regard perception and movement as a single unity; Husserl already spoke of a ‘constitutive duet’ of movement and perception, while more recently Alva Noë and others have developed a sensorimotor theory of perception based on phenomenological insights (Noë 2004; Thompson 2010). Additionally, perception affects our inner states as persons, for instance our emotional comportment. Perceiving an artwork might make one feel ecstatic or tranquil, while perceiving human terror can instill fear, stress or sadness. Reversely, our bodies and our thoughts also influence our perception (Merleau-Ponty 1962/2012, pp. 299–300). A person with negative thoughts sees the world in a different way than others. Likewise, attentional and affective modifications in the subject result in changes in the perceptual field as much as perception motivates and modifies our subjective modes of comportment. This makes sexual function and perception something that is highly individual, malleable, and fluid, and according to Gavin Rae, “intimately bound to the ambiguities of existence” (2020, p.180).

From a phenomenological viewpoint, ED also involves subjective perception. A healthy person may want to attain an erection when finding himself in a sexual situation that is pleasing to him. This sexual situation is usually not brought into reflective awareness (Moya and Larrain 2016); it is nevertheless something perceived (in a wide sense) by the subject, and such perceptions motivate bodily as well as intellectual responses. At the same time, his mind and body also influence his perception: if he is ‘not in the mood’, or if he is ill, the situation might not be transformed into a sexual spectacle. Phenomenologically speaking, sexual function should thus be considered in its concrete relations with (self-)perception, motivation, and bodily action (Moya and Larrain 2016).

To explore all these different components, the concept of sexual perception requires further clarification. Sexual perception is a type of perception that involves both subjective and objective modifications. Sexual perception might, for instance, induce feelings of desire and/or sexual arousal, as well as having certain effects on the body, e.g. an increased heart rate or erection (Janssen et al. 2008). Sexual perception also endows sexual sense to people, objects, and situations. Features of objects, such as colors, may enhance or feed into one’s sexual experience (Merleau-Ponty 1962/2012, pp. 216–223). This is in no matter restricted to visual perception. For instance, the familiar smell of one’s partner or certain kinds of music may stimulate sexual experience (Grammer et al. 2005; Georgiadis and Kringelbach 2012). Additionally, manual stimulation of the penis can induce erections and feelings of pleasure. At the same time, perception has the power to ‘destroy’ a sexual situation, for instance in perceiving objects or settings that entice fear.

According to Merleau-Ponty, the perceptual world is ‘animated’ (a process of sense-endowing of things experienced) in various ways, but also by “an Eros or Libido” (1962/2012, p. 158). The Eros or Libido enriches our perceptions with sexual connotations; it “gives external stimuli a sexual value or signification” (1962/2012, p. 158). Additionally, the Eros or Libido “sketches out for each subject the use to which he will put his objective body” (1962/2012, p. 158). Sexual perception is thus closely related to the subject and possible bodily action. In perceiving sexually animated things, the subject is simultaneously interacting with those things within an unfolding sexual situation: sexual perception involves a certain bodily intentionality, and the boundary between self and other is blurred (Rodier 2014).

Sexual perception and perception generally involves what Merleau-Ponty, to some extent following Heidegger, calls ‘projection’. To explain this concept, it is helpful to briefly consider a patient case Merleau-Ponty describes in his work (1962/2012, pp. 105–140; pp. 157–160). The case is about Schneider, a patient who is unable to perform actions on command or actions based on his own ‘inspiration’, but who is able to perform habitual actions within his immediate environment (1962/2012, pp. 105–115). Merleau-Ponty states that for an action performed on command, or in order to perform actions that are ‘new’ (i.e. not immediate, habitual reactions to the environment), we need to be able to project such new situations onto the world, since they are not yet there in the world (1962/2012, pp. 114–115). Waving at a person one sees is different than just waving into ‘space’, since when the actual person one wants to wave at is there in the world, one is invited by the world (i.e. the person) to wave; no extra projection is required. To wave into ‘empty’ space, on the other hand, requires an active projection of a world which solicits one’s waving action. Schneider, in Merleau-Ponty’s analysis, lacks this capacity.

Regarding sexual situation, Merleau-Ponty describes Schneider’s situation as follows:

Obscene pictures, conversations on sexual topics, and the perception of a body fail to arouse any desire in him. The patient hardly ever kisses, and the kiss has no value of sexual stimulation for him. Reactions are strictly local and never begin without contact. If foreplay is interrupted at that point, the sexual cycle does not seeks to be continued. During intercourse, intromissio is never spontaneous. If his partner reaches orgasm first and moves away, the nascent desire fades away. Things happen at each moment as if the subject did not know what to do. There are no active movements, except for a few instants prior to orgasm, which is itself quite brief. (1962/2012, p. 157).

The point to be taken from this passage is that our ability to project a sexual situation onto the world is essential to ‘normal’ sexual intercourse (1962/2012, p. 158). Sexuality for humans is not a simple habitual reaction, or a mere matter of stimuli and response. Successful sexual intercourse requires projections of sexual situations as well as the sexual animation of features of another person in ways that make them significant for our bodies, thereby inviting sexual action. Only given such successful projections will the body be able to react in a sexual manner towards a sexually laden world.

The function of projection, as a condition for sexual perception, provides a starting point in the development of a phenomenological aetiology of ED. Failure to successfully project sexual situations thus forms the basis for a phenomenological understanding of contemporary cases of ED. In subsequent sections we further develop this phenomenological aetiology, by introducing a set of conceptual tools borrowed from Merleau-Ponty’s work.

## A phenomenological etiology of erectile dysfunction

Schneider lacks the ability to project a sexual situation which alters his sexual perception and therefore his sexual action. We maintain that patients with ED could likewise have problems with their function of projection, although there are certainly exceptions. In ‘normal’ sexual perception, the sexual situation invites a subject to act, resulting in physical and psychological changes such as muscle tension, increased heart rates, erections, euphoria, and/or arousal (Janssen et al. 2008). In such cases, the subject has limited control over these changes. In the patient suffering from ED, physical changes are usually less pronounced (Bancroft and Janssen 2000; Dean and Lue 2005). It is also worth noting that in normal cases subjects are usually not as easily distracted when acting in the sexual situation, due to a phenomenon called ‘absorbed coping’ or ‘skillful coping’ (Dreyfus 2014). The patient suffering from ED, on the other hand, is more likely to feel uncomfortable in the sexual situation and is likewise more easily distracted. This phenomenon of distraction suggests that sexual projection in the patient with ED may not be of the qualitative kind which allows for successful absorbed coping, in other words, it may not be ‘strong’ enough. Several studies have suggested possible alterations in perception in patients with ED, which further suggests the importance of successful perceptual projection for absorbed coping (Cohen et al. 1985; Rosen 2001).

Since sexual perception generally affects the body and the mind in specific ways, and since the reaction to a situation we would typically deem ‘sexual’ is altered in the patient suffering from ED, a relationship between ED and subjective perception is plausible. Naturally, this does not establish any causal order; changes in perception and projection could occur after changes in erectile function. In this case, it is plausible that ED should have a strictly physiological cause, likely resulting in the complete impossibility of attaining an erection. However, if there is a possibility of achieving and maintaining an erection, as was also the case with Schneider, it is all the more remarkable that erection fails in what ‘normally’ appear to be sexual situations. In these cases, failure of attaining an erection ought to be partially attributed to psychological and/or social causes. One study suggests that only 18.6% of all patients with ED have a ‘complete ED’ (Chew et al. 2000). Further exploration of the relationship between ED, sexual projection, and perception is needed to shed light on the phenomenology of such psychological, social and bodily causes, which is what this section is devoted to.

First, it is necessary to explore in what ways sexual projection (and thus sexual perception and action) can be altered. So far, we discussed sexual projection within the context of a sexual situation. But over time, one’s capacity for sexual perception can change, just as one’s specific sexual preferences can change. We maintain that this could result from changes in one’s ability to project sexual objects and situations. Working with Merleau-Ponty’s philosophy, we propose that this ability or lack thereof is closely tied to three other phenomena: the ‘intentional arc’, ‘cognitive interference’, and perhaps most importantly the ‘body schema’. These will be discussed here in this order.

### The intentional arc

The first phenomenon we wish to relate to the function of sexual projection is the intentional arc. We take the concept of intentional arc to incorporate everything that determines a specific person’s outlook on the world: his past experiences, his future ideologies, his moral perspective, etc. (Merleau-Ponty 1962/2012, pp. 136–139). We can say that the intentional arc is at the basis of our very personal and social being; it defines the horizon of who we are, what we stand for, and how we actively comport ourselves toward our world.

The intentional arc is at the heart of the function of projection, since through our past experiences, our ideologies, our moral situation, and our current perceptual life, we relate ourselves to the world through conceiving of possible future actions. Past experiences of typing, a future goal of finishing an article, as well normative and factual beliefs pertaining to the practice of writing condition the projection of a computer keyboard as a typing mechanism serving a specific purpose. In short, our projecting of a context within a personal world of values, motives, and beliefs is at the background of the simplest motor actions such as in typing on a computer.

In the patient with ED, a motor action is lost, namely, the motor action of achieving an erection. We content that this loss of motor action can be due to a disturbance in the function of sexual projection, as previously indicated. But at the foundation of this sexual projection is, in turn, the intentional arc which allows us to project such situations. Merleau-Ponty affirms this, writing that “we will, all at once, discover sexual life as an original intentionality as well as the vital roots of perception, motricity, and representation, by grounding all of these ‘processes’ upon an ‘intentional arc’ that weakens for the patient and that for the normal subject gives experience its degree of vitality and fecundity” (1962/2012, p. 160). Merleau-Ponty concludes that the intentional arc in the patient case of Schneider is somehow modified, given that his function of projection is altered (as seen in his failure to perform ‘new actions’), and that the intentional arc is a fundamental component in the function of projection (1962/2012, pp. 135–137 and p. 160).

Due to changes in the intentional arc, the patient’s sexual projections change, and subsequently the way in which he is able to act with his body parts and other subjects around him changes (Dreyfus 2002; Merleau-Ponty 1962/2012, pp. 137–139). This is because all actions are performed against a certain perceptual background, and it is this very background that has changed due to changes in the intentional arc. To be sure, the intentional arc itself is not actively represented in either an intellectual or sensible fashion; it is rather a background operation. Nevertheless, it is intentional insofar as it determines meaningful comportment toward the world, and is not a part of the ‘unconscious’ in a Freudian sense. Therefore, if the intentional arc plays a role in forms of ED, it follows that these forms of ED cannot be traced back exclusively to physiological and/or psychoanalytic factors.

Past experiences as well as future plans and ideologies thus determine sexual projection and thereby also one’s bodily motivations and actions. It goes without saying that this holds in particular for past hostile (sexual) encounters. But future plans or tacitly projected expectations may also play pivotal roles. For instance, seeing no long-term future with one’s sexual partner might contribute to failure of sexual projection, just as expectations of terror or fear, whether justified or not, can make sexual projection impossible for a subject. Here it can be seen that the results of our additional phenomenological aetiology correspond to findings from psychology.

### Cognitive interference

We have stated before that a situation, sexual or otherwise, requires absorbed coping. We are all familiar with the phenomenon of distraction, where phenomena interfere with what is our preferred focal object. Distraction can mean simply an undesired shift of attention toward another object, but it can also involve the ‘destruction’ of a whole situation. For instance, one might shift perceptual attention to one’s phone in the course of sexual intercourse, as a result of which the phone now ‘lights up’ in one’s perceptual field which distracts from intercourse. But it is also possible for shifts of attention to entirely break down a situation, even when the object of attention is undetermined. To give a mundane example of this: one might fail to urinate on the mere expectation that an indeterminate ‘someone’ might enter the bathroom.

Empirical research on psychological characteristics of ED suggests distraction happens more often in patients with ED (Barlow 1986). In like fashion, Merleau-Ponty points out that “the majority of impotent subjects ‘are not immersed in what they do’” (1962/2012, p. 159). Such distracting thoughts can be compulsive and unfree and need not pertain to any specific objects. Just like the philosopher is sometimes caught out by a thought “at any street corner” (Camus 1955/2013, p. 10), which in turn alters the way he projects or represents the world (Camus 1955/2013, pp. 12–13), the person suffering from an ED might be caught out by a thought or a distraction in the sexual situation. This thought or distraction draws him out of the lived situation, thereby losing the ability to sexually project, and unwillingly enters another ‘space’, whether of anxiety or something else, a world in which an erect penis does not have immediate signification.

The way thought deconstructs the sexual situation deserves further elaboration, since it could be essential to clarifying the subjective processes involved in many cases of ED. In Merleau-Ponty’s phenomenology, the body is considered as unified, and the unification of the body is held to be necessary in order to function properly (Merleau-Ponty 1962/2012, pp. 149–155). Consider the case in which someone were to pick up something from the ground. This may appear to consist simply of a person noticing the object with his eyes and a subsequent grasping of the object with the hand. Closer examination, however, reveals the involvement of the whole body. Picking something up from the ground is highly orchestrated movement involving the entire body: the feet are put slightly apart, knees are bend, the back curved, the body keeps its balance with the contralateral arm, the head directs towards the object, the fingers open and close, etc.

Although easily overlooked, the body is also unified in the sexual situation (Moya and Larrain 2016). It is not merely the penis that becomes rigid and enlarges; the whole body position alters in a habitual manner, as can also be observed in sexual positions. Sexual intercourse likewise does not depend solely on the ability of a penis to penetrate: it is in effect a complexly orchestrated movement of two bodies of two individuals. The sexual situation, at least during sexual intercourse, is thus a social situation, requiring the whole body to adapt in ways appropriate to the particular situation (Spinelli 1997).

Thought can deconstruct the unity of the body, and this might be one of the mechanisms in which patients with ED deconstruct their sexual projection. This can be enlightened by an example: when a thought enters the sexual situation that is related to the function of the penis, as happens often in patients suffering from an erectile dysfunction (Barlow 1986; Nobre 2010), focus may shift towards the performance of the penis. In this manner, the skillful coping of the body as a unity may be partially deconstructed. Similar ideas can be found in *Phenomenology of Perception*, where Merleau-Ponty recognizes that erotic perception aims “through one body […] at another body, and it is accomplished in the world, not within consciousness” (1962/2012, p. 159). He concludes that “a scene does not have a sexual signification when I imagine, even confusedly, its possible relation to my sexual organs or my states of pleasure, but rather when it exists for my body” (1962/2012, p. 159). In other words, absorbed coping peculiarly requires *not* being conscious of what one does; it requires a skillful and absorbed intentionality of the body as a whole.

Merleau-Ponty also notes that “for the normal person, a body is not perceived merely as just another object” (1962/2012, p. 158). Patricia Moya and Maria Elena Larrain also confirm that in Merleau-Ponty’s phenomenology, “one’s own body and those of others are not perceived as objects, nor as causes or instruments, but rather as expressions of personal life and of relation with others” (2016, p. 754). Thought, however, by its nature objectifies, and thereby it potentially deconstructs the lived situation, especially when the situation relies on pre-reflective motor action. For the patient with ED, one’s own body might be perceived as another object, which results in the deconstruction of the unity of the body that is presupposed in living sexual situations. This objectification is also significant in solitary sexual activities, such as masturbation: Ernesto Spinelli points out that even during masturbation, the other body or person is present in an imaginary or internalized way (2013).

We can reconnect these analyses of distracting thoughts and sexual projection to the concept of the intentional arc. As the previous section elaborated, sexual experience is affected by future plans, past experiences, and a variety of other things that the concept of intentional arc captures. Recurring thoughts also feed into this intentional arc; their effects may become habitualized and thereby a part of the intentional arc (which consisted partially of habitualized past experiences). This way, such thoughts may subsequently exert their effect on future sexual encounters. The thus reconfigured intentional arc can then make such recurring thoughts more likely, which again feed back into the intentional arc, etc. This downward spiral can also be found in the biopsychological model (Yafi et al. 2016), but lacking phenomenological underpinnings.

### Body schema

In the previous subsections, we have found that the intentional arc, thoughts and distraction are able to change a person’s ability to project a sexual situation. This alteration of the ability to project will cause a change in perception, and therefore a change in the background to which a person can respond with his body. In the patient suffering from ED, the projection is changed in such a way that the alteration or loss of sexual perception results in the inadequacy of acquiring or maintaining a penile erection for satisfactory sexual activity. At this point, one could say that if the sexual projection of the patient were to be restored, the erectile function would be restored too. However, this need not be the case. In this subsection, we will enquire into a way in which the ED might persist, even in patients where sexual projection is restored. We will do this by appealing to another concept of Merleau-Ponty, namely, the body schema.

The body schema refers to a kind of pre-reflective awareness of both the capacities and the whereabouts of one’s own body (Merleau-Ponty 1962/2012, pp. 100–105). For instance, one knows, in a bodily sense, where one’s arms are, even if one do not consciously think about where they are. Likewise, one knows how to use one’s body to walk or pick up something from a table without having to think about it. The concept of body schema therefore involves a type of self-knowledge or subjectivity, but prior to reflection (Merleau-Ponty, 1962/2012, p. 146). Substantial work has already been done on the body schema and its role in medicine as well as other sciences, perhaps most notably by Shaun Gallagher.^2^

Any alterations in the body schema involve alterations in one’s sense of self, meaning one’s understanding of one’s motor capacities as well as one’s capacities to project situations. For instance, the stick used by the old man becomes a part of his body schema. If he were to fall, his whole body would move in a different way with the stick, because the stick affects the pre-reflectively understood unity of his body. Moreover, it allows him to project situations he was previously incapable of, such as venturing longer or more risky walks. The world thus solicits different types of actions for him.

The body schema can, however, expel body parts too. Although Merleau-Ponty’s treatment of it can no longer be regarded as accurate, the phenomenon of the phantom limb illustrates that the body schema may persist in presenting a body part that’s no longer there (Merleau-Ponty 1962/2012, pp. 82–85 and pp. 100–105). In the patient suffering from anosognosia (e.g. inability to feel an arm that is there), there is a body schema defect in which the body schema has expelled the very arm, even though the arm is still there (Merleau-Ponty 1962/2012, p. 102). In similar ways, we propose that the sexual projection defect of the patient suffering from ED may involve expulsion of the penile erection in the body schema.

The patient suffering from ED has ‘lost’ the workings of his erect penis. We suggest that this loss may be effectuated first by changes in sexual projection, which after a sustained period of time may result in persisting changes in the body schema. The body habitually ‘forgets’ that the penis can achieve an erect state. The body schema thus effectively neglects the working of the penis, as it grows accustomed to having only a flaccid penis. It is easily imaginable that even upon the return of proper sexual projection and sexual immersion, such a habituated body schema may persist—comparable perhaps to the way a phantom limb may persist long after the real limb’s removal. The aetiology of the disorder might therefore shift: while it is initially brought on by distracting thoughts, doubts, anxieties, or simply by past experiences or future expectations which become habituated in the intentional arc, the dysfunction eventually comes to persist in the pre-reflective body schema.

## Existential implications

We have explored the relationship between the workings of the penile erection, sexual perception, sexual projection, the intentional arc, distracting thoughts, and the body schema. Alterations within any of these interrelated components affect the way one perceives and comports oneself toward the world. In this section, we consider how the feelings and experiences of such alterations may further contribute to the development of ED. The ‘existential situation’ of the patient suffering from ED may thus affect the course of the disorder itself. This is recognized by the current biopsychosocial model of ED (Yafi et al. 2016), but not on the basis of a phenomenological and existential approach.

We have suggested that certain types of ED can become a persistent problem within the habitual body schema. This meant that sexual projection might return on grounds of changes in the intentional arc, but that ED might nonetheless persist, given that the body schema has unlearned the erectile function of the penis. Therefore, we wish to briefly discuss the existential situation of two types of patients here. First, a patient with a failure of sexual projection (with or without a failure in the body schema). Second, a patient with a failure of the body schema but with a recovered sexual projection.

### Projection in the social environment

The severity of the absence of sexual projection is of course, patient-determined. We will consider some general remarks and discuss a few ways in which the failure of sexual projection might alter the patient’s existential situation and his self-understanding.

One way in which failure of sexual projection influences an individual’s existential situation concerns the social sphere. The ED patient may no longer be struck by the aesthetic or erotic appearance of another person; social interaction generally may become partially dormant. Friends might be occupied with gazing at beautiful persons, talking about these persons, or trying to chat up with them. This affects a subject’s social situation. One patient says the following about his erective problem: “Outwardly, it always seemed that I was one of the lads and that I was okay … but inside … I didn’t feel that I was matching up to them” (Tomlinson and Wright 2004, p. 1038). Another says: “you have all those jokes in the showers after golf or football or whatever, […] but beyond that it’s [the erectile dysfunction] not a subject that is discussed” (Pontin et al. 2002, p. 269). In these and other ways, ED may affect one’s sense of self-worth, social standing and/or invoke negative feelings, aspects that are also recognized by the biopsychosocial model (Yafi 2016). Based on our framework, it can be seen that these aspects might affect the intentional arc, potentially leading to a worsening of the disorder.

Social problems can also exist within one’s sexual relationship. Two patients state that they cannot or do not want to talk about their sexual problems with their wives; another patient remarks he has recurring thoughts that his wife is cheating on him; yet another feels frustrated that he cannot satisfy the ‘needs’ of his partner (Pontin et al. 2002, pp. 267–270). Another patient, in reflecting on the way the disorder altered his social being and relationships with others, exclaimed: “I can’t live like this. […] You’re [talking about himself] the only one in the world” (Tomlinson and Wright 2004, p. 1038). Relationship problems like these are recognized as a frequent problem of ED according to the biopsychosocial model (Yafi et al. 2016).

### Altered penile body schema

In the patient who has retrieved (some) sexual projection but who fails to achieve or maintain an erection due to the embeddedness of the problem in the body schema, the existential situation is quite different. This is because in the patients of the previous subsection the sexual situation does not solicit sexual action, since they fail to project a sexual situation in the first place. The patient with the altered penile body schema and a preserved sexual projection is, by contrast, invited by the sexual world. However, he fails to respond to this world.

Merleau-Ponty states in the case of a lost limb that “manipulable objects, precisely insofar as they appear manipulable, appeal to a hand that I no longer have” (1962/2012, p. 84); this can be translated to the case of ED. This is because the sexual situation now appeals to a penis that one no longer “has”, i.e. one can no longer act upon solicitations from the world as one would want to.

This failure has several implications for the patient. First, there is a sense in which the disorder cannot be genuinely understood by the patient himself: “To understand is to experience the accord between what we aim at and what is given, between the intention and the realization” (Merleau-Ponty 1962/2012, p. 146). The intention of the patient is of course, to have an erect penis and to engage in sexual practice, and the world also invites him to do so. However there is an incongruity in this call from the world and the inability to act upon it. The feelings and circumstances are there, but the desired actions cannot be actualized. One patient exclaims “I can’t really put a finger on it”, and “other men are mystified” (Pontin et al. 2002, pp. 266–267). These feelings of uncertainty and doubt doubtlessly contribute negatively to the well-being of the patient.

Second, most men attribute manhood to the workings of the penis (Barlow 1986; Nobre 2010). Because an erection is not embedded in the body schema anymore, and yet they are being invited by their sexually projected world, a patient might feel that he has lost his manhood. One study describes that the patients with erectile dysfunction rank having an erection as the most important sign that confirms and confers manhood (Pontin et al. 2002, p. 266). Another patient exclaims: “you are not a man if you can’t get an erection … nobody’s going to have any respect for you” (Tomlinson and Wright 2004, p. 1038), and the study furthermore states that “the most common initial reaction to erectile dysfunction was a sense of emasculation” (Tomlinson and Wright 2004, p. 1038). This, too, contributes negatively to the well-being of the patient.

Third, sexual situations becomes something to be avoided, just like the patient with multiple sclerosis wants to avoid the stairs: it involves too much struggle and confrontation (Toombs 1995, p. 11). One patient suffering from ED mentions that “if I was to walk out of here and meet a drop dead gorgeous women … [I’d] think “Well, it’s pointless chatting her up, because I can’t do anything” (Tomlinson and Wright 2004, p. 1038). It is not difficult to imagine how such avoidance behavior may elicit negative feelings and low self-esteem.

Finally, it is worth repeating that these existential consequences of ED in turn affect the disorder itself. Negative feelings, beliefs, self-worth, etc. become part of the patient’s intentional arc. They are reintegrated into the horizon from which the subject projects meaning onto the world, changing how the patient perceives and copes with future sexual encounters. The disorder might thus worsen overtime, especially without proper treatment.

## Practical implications

Besides giving the biopsychosocial theory of ED more phenomenological and existential depth, various practical implications follow from our phenomenological analysis. In this section, several of these implications for the scientific and practical fields regarding the care for patients suffering from ED will be discussed. We hope that by briefly elaborating on these implications, future research endeavors might improve the science, the philosophy, and the medicine concerning ED.

### Doctor–patient relationship and diagnosis

As mentioned earlier, denial and embarrassment are common among ED patients and may prevent them from seeking treatment. This may indicate that the doctor–patient relationship is compromised and that current forms of treatment do not sufficiently accommodate the existential situation of the patient. Likewise, most research focuses on the workings of the penis, the objective body, the conscious mind and its relation to the sexual situation, but not so much on the existential situation of the patient.

Our analysis hopes to restore some of the existential components of suffering from ED. Phenomenological analysis reveals that not only the objective workings of the penis and the mind matter, but also the patient’s whole experience of himself and the world. Perception modifies the projection of the sexual situation, and in some patients, as we have seen, their sense of manhood can also be broken down (Tomlinson and Wright 2004, p. 1038). We proposed that past negative experiences can become integrated in the intentional arc, leading to a worsening of the disorder. Phenomenology shows that the experience of the patient is paramount to get a grip on the patient’s ED. This is because the patient’s past experiences, the experience of the ED itself, and the experience of the current environment by the patient all orchestrate themselves within the intentional arc, thereby determining penile functions.

A phenomenological account can help to restore subjectivity to the patient, by focusing on what really matters: the experience of the patient. Patients suffering from ED are vulnerable patients, and the objectifying attitude of the current medical paradigm which often solely focuses on bodily workings might be unhelpful in medical consultation and the physician’s ability to understand and help the patient. Our phenomenological approach, combining the unity of the body, perceptual projection, the intentional arc, the body schema, and experience can successfully restore subjectivity to the ED patient, thus accommodating a better understanding of the phenomenon of ED and diminishing the existing ‘taboo’ around ED.

Even though empirical evidence is lacking, some writers suggest that the prevalence of ED and interest in ED has been partially dependent on the introduction of sildenafil (Hart and Wellings 2002; Moreira Jr et al. 2002). This is because the availability of treatment might lead to the medicalization of phenomena otherwise deemed normal, which seems to be an expression of the contemporary tendency to (pharmacologically) treat disorders seen as physiological dysfunctions. As already mentioned, our phenomenological approach tries to show that the diagnosis of ED is highly subjective, based on the patient’s perception, experience and social environment. Our approach is therefore able to explain the wide variance in prevalence of ED and the way the availability of treatment (altering future goals, a component of the intentional arc) might change the way the disorder is perceived, while physiological etiologies partially fail to explain these dynamic components contributing to the diagnosis of ED. A phenomenological approach can thus strengthen contemporary thought in providing explanations for these important and puzzling aspects of ED.

A phenomenological approach might also strengthen other aspects of the biopsychosocial model of ED. Although it is primarily theoretical and deals exclusively with the first-person perspective, elements of a phenomenological framework can nonetheless be considered in the offices of physicians. Physicians can put phenomenological theory into practice by asking about the experience, occurrence, the nature, and the associated problems of ED in such patients. We expect that if physicians focus such conversations on the patient’s past experiences, the patient’s experiences of the environment, the way the patient experiences his ED, and the patient’s overall existential situation, patients will be more willing to discuss ED with physicians, thereby both improving treatment and increasing the number of ED patients with professional treatment. Therefore, our theoretical framework especially complements the psychological and social aspects of the biopsychological model of ED, aspects that are often overlooked during treatment of ED patients (Carpiano 2001).

Our phenomenological account can eventually also be helpful in the diagnosis of several (phases) of ED. As we have seen, thoughts may be able to influence the current (sexual) projection of the patient, the intentional arc in turn influences projection, and the body schema reifies flaccid functioning of the penis. Diagnosis and treatment of ED can rely on enquiry into these components of perception.

A first step regarding a patient presenting himself with an ED could involve exploring the field of sexual perception. Sexual perception can be explored when a physician asks questions about whether the patient ‘adds’ sexual connotations to otherwise normal situations. Some questions could be about whether he (1) is sexually attracted to people in normal daily situations, (2) fantasizes about sex, (3) dreams sexually, or whether he (4) has sexual impulses. A physician might make use of certain questionnaires or tools for these investigations, such as the Sexual Desire Inventory (SDI) (Spector et al. 1996). If patients answer negatively to these kind of questions, or scores low on the SDI, this may indicate a structural problem in sexual projection. It could also be that the patient answers positively to the questions posited above, but that he reports a sudden loss of sexual desire in a sexual situation. This might suggest, based on our framework, that the patient’s sexual projection is ‘broken down’ in a situation previously deemed sexual. A high score on the SDI and/or positive answers to the questions posited above, without a loss of sexual desire in a sexual situation, may indicate a body schema defect. In all cases, it is worthwhile to explore contributing factors in the patient’s intentional arc, such as past sexual encounters, the status of the patient’s current relationship, drug use, the person’s view on his sexual life, partner perspectives, future goals in the field of sexuality, anxiety, sexual confidence and self-perception.

We venture to say that if sexual perception is found to be deconstructed suddenly, the physician might start by exploring the patient’s cognitive situation and ask about the patient’s thoughts and distractions in the sexual situation. In this way, it is possible that a clearer etiology of the patient’s ED can be found and this can form the basis for future treatment options. If according to our framework, the problem is found in the body schema, the physician might set forth an enquiry into the personal history of the patient and his sexual projection. If there was a modification in sexual perception before the ED took place, there could be an intentional arc and/or thought/distraction problem prior to the body schema problem. Even though these claims necessitate more empirical research, it is possible that the physician might eventually be able to better distinguish between the ED’s psychological, social and physiological components according to a phenomenological theory of ED.

### Treatment

By focusing on patient perception, treatment might be more personally tailored. If empirical findings of physicians match our theoretical considerations about the body schema, the physician might search for ways in which to alter the body schema. We considered the idea that the body schema might unlearn the workings of erections. Perhaps, the erection can be ‘relearned’ or reinforced by several therapies. One of these therapies could be a penile implant, a device that is used to bring the penis in an erect state. Theoretically, this might aid the body schema in its erective movements. Additionally, a similar positive effect may result from the use of PDE5 inhibitors—medications which can induce erections in men with ED. In some patient groups, the spontaneous erectile function has been restored after administration of PDE5 inhibitors (Son et al. 2004).

On the other hand, if empirical findings match our theory of the absence of sexual projection, a physician might be reluctant to adopt an approach that consists of medications and/or implants, since according to our framework, there is a possibility that the function of erection can be restored by a restoration of sexual projection. The doctor could find out the possible cause for the change in sexual perception, which can be either structural or a quick collapse (due to thoughts or distractions). Treatment can then consist of psychological interventions with the aim of restoring the patient’s sexual projection. In structural perceptive problems, we suggest that exploring and resolving issues in the patient’s past, the patient’s goals and the patient’s current situation might be helpful. In patients where sexual perception suddenly collapses, therapy might involve exploring the patient’s thoughts during the sexual situation as well as ways in which the patient can break the patterns of such thoughts. In such cases referral to a psychologist or sexologist can be advisable; recent studies indicate the benefit of cognitive behavioral therapy and other psychological interventions for patients suffering from ED (Khan et al. 2017; McCabe et al. 2008).

## Conclusion

The aim of this article was to introduce a new phenomenological framework for investigating ED to contribute to existing physiological and psychosocial thought concerning ED. Most contemporary research focuses on psychosocial components and/or the physiological basis of ED, leaving the subjective field of perception insufficiently explored. While a subjective approach can never offer a viable treatment method purely on its own, we believe investigating the subject’s viewpoint opens up new, interesting, and valuable ways of looking at the patients’ (existential) situation and that it may positively affect the way patients are approached, diagnosed and treated. Based primarily on the work of Merleau-Ponty, we suggested ED may have its origins, subjectively speaking, in modifications in sexual projection, the intentional arc, distracting thoughts and finally the body schema. By incorporating the way the patient sexually perceives the world, his partner(s) and himself, more insight can be gained into the origin of the problem of ED in patient specific cases, which can translate into better future diagnosis and treatment, as well as a better patient self-understanding.

## Footnotes

1 This does certainly not imply that other phenomenological strands of thought are less applicable to sexuality and ED. In her recent book (2019), Susi Ferrarello presents a very interesting phenomenological study of sex, love and intimacy based on Husserl’s phenomenology. Additionally, several authors have studied Sartre’s work in relation to sexuality (Lapointe, 1974; Mirvish, 1994; Burr, 2003; Pearce, 2011).

2 Gallagher, in his important book *How the body shapes the mind* (2005), argues that our embodied existence contributes to our consciousness, communication, and to our interaction with the world. Like us, he uses the phenomenological concept of the body schema as a crossing between phenomenological and empirical studies – although he does not use it to reinforce the biopsychosocial approach of ED.
